# Measles resurgence in Texas: a public health wake-up call

**DOI:** 10.1097/MS9.0000000000003849

**Published:** 2025-09-12

**Authors:** Erum Siddiqui, Muhammad Saad Khan, Devya Khaim Chandani, Maliha Khalid, Aminath Waafira

**Affiliations:** a Department of Medicine, Jinnah Sindh Medical University, Karachi, Pakistan; b Department of Medicine, Shaheed Mohtarma Benazir Bhutto Medical College (SMBBMC), Karachi, Pakistan; c School of Medicine, The Maldives National University, Malé, Maldives

**Keywords:** immunization policy, measles resurgence, public health outbreak, vaccine

## Abstract

Measles, a highly contagious viral disease once nearing elimination in the USA, has resurged, with Texas reporting its most severe outbreak in nearly three decades. As of February 2025, over 48 confirmed cases, predominantly among unvaccinated children, highlight critical vulnerabilities in state immunization policy. Factors driving this resurgence include declining vaccination rates, widespread misinformation, legislative allowances for nonmedical exemptions, and public health disruptions from the COVID-19 pandemic. The Texas outbreak reflects a global trend, with measles cases increasing by nearly 80% in 2023 compared to pre-pandemic levels, underscoring gaps in herd immunity and the fragility of elimination gains. This article examines epidemiological features, transmission dynamics, and clinical manifestations of measles, emphasizing the severe complications and public health burden it poses. It also reviews preventive measures, ethical considerations surrounding vaccine mandates, and strategies for improving coverage, including restricting exemptions, expanding rural outreach, and combating misinformation. Without immediate, sustained interventions, Texas risks recurring outbreaks, threatening broader national measles control efforts and reversing decades of progress in vaccine-preventable disease prevention.

## Introduction

Measles, once nearly eradicated in the USA, is making a troubling comeback. The current Texas outbreak serves as a warning about what might occur when vaccination rates decline, public health awareness wanes, and misinformation spreads unchecked. The increase in incidence necessitates immediate measures to enhance long-term immunization strategies and protect herd immunity. Neglecting vaccination promotion poses a significant risk of reversing decades of medical progress. It is not an isolated event but rather part of a larger global trend that has major public health implications. This manuscript has been developed in accordance with the TITAN Guidelines (2025)^[1]^.

## Understanding measles: a highly contagious threat

Measles is a viral infection caused by the measles virus (MeV), a member of the Paramyxoviridae family^[2]^. It is one of the most contagious illnesses known to humans, with a basic reproduction number (*R*₀) ranging from 12 to 18. This means that a single infected individual can transmit the virus to 12–18 others in a non-immune population^[3]^. Transmission occurs via respiratory droplets, rendering densely populated settings like schools, daycare facilities, and public transit especially susceptible to outbreaks^[4]^.

The clinical progression of measles initiates with a prodromal phase, marked by high fever, cough, conjunctivitis, and coryza^[5]^. Koplik’s spots subsequently emerge as small white lesions on the buccal mucosa and are considered pathognomonic for measles^[6]^. A typical maculopapular rash spreads from the face downward within days, indicating a severe disease^[7]^. Although most cases resolve without complications, measles poses a considerable risk of serious consequences, such as pneumonia, encephalitis, blindness, and mortality^[8]^. Individuals under 5 years of age, pregnant women, and immunocompromised individuals are at the greatest risk. The most feared long-term outcome of measles is subacute sclerosing panencephalitis, a rare yet lethal neurological disorder that may arise years post-infection^[9]^. In light of these concerns, the importance of immunization is paramount.

## The global measles resurgence: a disturbing trend

Efforts to eliminate measles have mainly been successful, with global vaccination initiatives reducing mortality by 78% between 2000 and 2008^[10]^. Nonetheless, the disease is experiencing a concerning resurgence globally, with cases increasing by almost 80% in 2023 relative to pre-pandemic levels, according to reports from the WHO and CDC^[11]^. This return is driven by various factors, including vaccine reluctance fueled by misinformation and distrust, which is driving many parents to delay or refuse immunization. The COVID-19 pandemic further disrupted routine vaccination programs, leading to significant immunity gaps^[12]^. Additionally, political and social instability, including conflicts, refugee crises, and economic hardships, has strained healthcare systems, making vaccines less accessible in vulnerable regions. Many countries have underfunded immunization efforts, and even high-income nations are struggling to restore vaccination rates to pre-pandemic levels due to a weakened public health infrastructure. The misconception that vaccine-preventable illnesses are no longer a significant danger has caused complacency in developed nations, reinforcing the risk of outbreaks and emphasizing the need for sustained high vaccine coverage.

## The Texas outbreak: a crisis in the making

As of February 2025, Texas has documented more than 48 confirmed cases of measles, predominantly among unvaccinated children and young people. Although rural counties, such as Gaines, have historically had low immunization rates and have been the most severely affected, major metropolitan areas like Houston and Dallas have also reported cases, raising concerns about urban transmission. Health authorities are endeavoring to manage the outbreak; however, the situation has revealed flaws in the state’s public health regulations. This represents Texas’s most significant epidemic over the past three decades and may deteriorate without prompt intervention^[13]^.

A primary factor is the increase in nonmedical vaccination exemptions. Texas permits parents to decline vaccinations for religious or philosophical reasons, with over 80,000 exemptions documented in the previous academic year^[14]^. Conversely, California banned nonmedical exemptions in 2016, resulting in a 3.3% rise in MMR vaccination coverage and a 2.4% decline in nonmedical exemptions. In 2019, New York eliminated religious exemptions, leading to increased immunization rates. These examples indicate policy mechanisms that Texas could implement to strengthen coverage^[15]^.

Texas remains vulnerable because of legislative barriers to stricter mandates and persistent anti-vaccine sentiment^[15]^. Past outbreaks in 2013 and 2019 were linked to low immunization rates. Weak enforcement further amplifies risk, leaving Texas more susceptible than states with robust vaccination policies.

Social media and advocacy groups like Texans for Vaccine Choice, which are against mandates, have also spread false information that has caused people to be hesitant. False claims, such as the debunked MMR–autism link and exaggerated risk narratives, have spread widely, while the COVID-19 pandemic eroded trust in public health and disrupted routine immunization. The initial response in some communities was slow, and incomplete immunization records hampered contact tracing, allowing the virus to circulate longer than it should have.

## Treatment options for measles

While no particular antiviral treatment for measles is available, supportive care is essential to relieving symptoms and preventing complications. The WHO recommends vitamin A supplementation for all children diagnosed with measles, as it significantly reduces morbidity and mortality, especially in malnourished children. Antipyretics such as acetaminophen or ibuprofen mitigate discomfort, but adequate hydration and rest are essential for recovery. Antibiotics may be required to address subsequent bacterial infections, including pneumonia or otitis media. Severe cases, notably those with complications such as pneumonia or encephalitis, sometimes require hospitalization for intense supportive care, including oxygen therapy and intravenous fluids. Despite these treatment options, vaccination remains the most effective strategy for preventing measles.

## Preventing future outbreaks: a call to action

Preventing future measles outbreaks requires a comprehensive public health response. Texas should curb nonmedical exemptions and adopt conditional school-entry requirements with periodic audits of medical exemptions. Expanding community-based immunization programs, particularly in rural areas with low vaccine coverage, and deploying mobile vaccination clinics can improve accessibility.

Mandates provoke ethical dilemmas regarding autonomy and parental fears about adverse impacts. Clear risk communication, transparent safety control, and credible spokespersons like pediatricians, religious leaders, and school nurses can mitigate these issues. Informed-consent systems and transparent appeal processes can safeguard individual rights while emphasizing community protection. Misinformation must be addressed appropriately. Boosting surveillance, promoting equitable vaccine access, and addressing local barriers to uptake are crucial for efficient control.

## Misinformation analysis

Social media amplifies false vaccine claims closely associated with the Texas outbreak. Groups such as Texans for Vaccine Choice have spread debunked theories, including the false MMR–autism link. Algorithms that favor sensationalism make it easier for misinformation to propagate than for corrections. Public health organizations must partner with technology firms to identify misinformation, disseminate evidence-based information, and execute prompt fact-checking responses.

## Ethical considerations in vaccine mandates

Stricter vaccination requirements raise more ethical concerns about striking a balance between protecting the public’s health and preserving individual liberty. Although mandates have been shown to increase immunization rates effectively and reduce outbreaks, they may be met with resistance if they are perceived as violating one’s liberty. Policymakers should consider hybrid approaches that uphold public health priorities while respecting individual rights. To promote acceptance, it is crucial to communicate openly and have an ongoing discussion with communities.

## Key recommendations

- Abolish non-medical vaccine exemptions to boost coverage.
- Promote rural immunization and mobile clinics.
- Collaborate with social media sites to prevent the spread of misinformation.
- Establish transparent vaccine safety monitoring.
- Adopt conditional school entry requirements.
- Enhancement of disease surveillance for early detection.
- Regular targeted public health programs.

## Conclusion

The resurgence of measles in Texas is an urgent reminder to public health officials, policymakers, and the general public. It highlights the consequences of declining immunization rates and the risks of misinformation. Although vaccines have successfully prevented measles for decades, gaps in coverage have allowed this highly contagious disease to reemerge. The Texas outbreak must be viewed as a watershed moment, an opportunity to reiterate the significant role of immunization, contribute to more substantial public health infrastructure, and adopt measures to forestall similar crises in the future.

## Data Availability

No data were generated for this manuscript.

## Research registration unique identifying number (UIN)

Name of the registry: N/A. Unique Identifying number or registration ID: N/A. Hyperlink to your specific registration (must be publicly accessible and will be checked): N/A.
